# The genome sequence of the Shoulder-striped Wainscot moth, *Leucania comma *Linnaeus, 1761

**DOI:** 10.12688/wellcomeopenres.23326.1

**Published:** 2024-11-05

**Authors:** Stephanie Holt, Laura Sivess, Inez Januszczak, Gavin R. Broad, Chris Fletcher, Denise C. Wawman, Natural History Museum Genome Acquisition Lab, University of Oxford and Wytham Woods Genome Acquisition Lab, Darwin Tree of Life Barcoding collective, Wellcome Sanger Institute Tree of Life Management, Samples and Laboratory team, Wellcome Sanger Institute Scientific Operations: Sequencing Operations, Wellcome Sanger Institute Tree of Life Core Informatics team, Tree of Life Core Informatics collective, Darwin Tree of Life Consortium

**Affiliations:** 1 Natural History Museum, London, England, UK; 2 University of Oxford, Oxford, England, UK

**Keywords:** Leucania comma, Shoulder-striped Wainscot moth, genome sequence, chromosomal, Lepidoptera

## Abstract

We present a genome assembly from an individual female *Leucania comma* (the Shoulder-striped Wainscot moth; Arthropoda; Insecta; Lepidoptera; Noctuidae). The genome sequence spans 751.70 megabases. Most of the assembly is scaffolded into 32 chromosomal pseudomolecules, including the Z and W sex chromosomes. The mitochondrial genome has also been assembled and is 15.37 kilobases in length. Gene annotation of this assembly on Ensembl identified 12,477 protein-coding genes.

## Species taxonomy

Eukaryota; Opisthokonta; Metazoa; Eumetazoa; Bilateria; Protostomia; Ecdysozoa; Panarthropoda; Arthropoda; Mandibulata; Pancrustacea; Hexapoda; Insecta; Dicondylia; Pterygota; Neoptera; Endopterygota; Amphiesmenoptera; Lepidoptera; Glossata; Neolepidoptera; Heteroneura; Ditrysia; Obtectomera; Noctuoidea; Noctuidae; Hadeninae; *Leucania*; *Leucania comma* Linnaeus, 1761 (NCBI:txid987968).

## Background

The Shoulder-striped Wainscot ( *Leucania comma*) is a relatively widespread species, although records on the National Biodiversity Network Atlas demonstrate that it becomes increasingly infrequent towards the north of England and into Scotland, although the most northerly record is close to Beridale on the northeast coast of Scotland, with several records occurring as far north as Inverness ( NBN Atlas Partnership, 2024). It is listed as a UK Biodiversity Action Plan species and as a Species of Principle Importance (England) under the Natural Environment and Rural Communities (NERC) Act 2006 Section 41, as a Species of Principle Importance (Wales) under the NERC Act 2006 Section 42, is included on the Scottish Biodiversity List, and is a Northern Ireland Priority Species ( NBN Atlas Partnership, 2024).

The species occurs in a wide range of habitats, including fens, marshes, grasslands, gardens, and damp woodlands ( Wall, 2024). The adult forewing is clearly marked with a long black streak running from the base to the centre of the wing, a white costal streak, and clear white veins running from the centre of the wing to the termen against a background of greyish-brown or pale straw ( Waring *et al.*, 2017). The adult has a wingspan of 34–40 mm and a forewing length of 16–19 mm and a single flight season of May to July ( Lewis, 2018). The larvae feed on grasses, and are particularly associated with *Deschampsia flexuosa* and *Festuca sp.* ( Robinson *et al.*, 2023).

This specimen was captured in a light trap at the Gilbert White House & Museum in Selborne, near Alton, Hampshire, during a genome-blitz for the Darwin Tree of Life project by a team from the Natural History Museum. Gilbert White (1720–1793) was a pioneer in observational natural history and commonly held to be the ‘father of ecology’. He is famed for his *Natural History and Antiquities of Selborne* ( White, 1789), which highlighted the depths of his studies in his home village, particularly in his garden from which this specimen was taken. In Hampshire in general this species remains widespread ( Wall, 2024), however on this site it remains a sporadic visitor to the light traps.

## Genome sequence report

**Figure 1.**
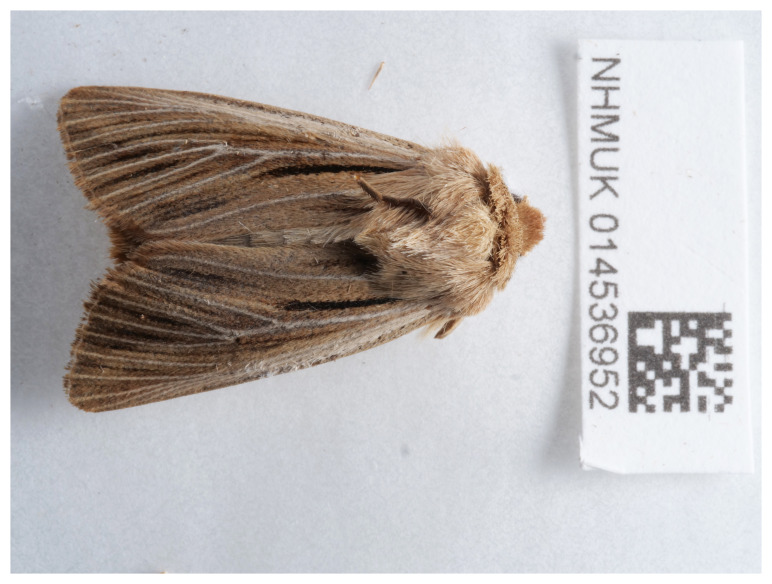
Photograph of the *Leucania comma* (ilLeuComm1) specimen used for genome sequencing.

The genome of an adult female specimen of *Leucania comma* ( Figure 1) was sequenced using Pacific Biosciences single-molecule HiFi long reads, generating a total of 25.90 Gb (gigabases) from 2.13 million reads, providing approximately 34-fold coverage. Primary assembly contigs were scaffolded with chromosome conformation Hi-C data, which produced 94.86 Gb from 628.22 million reads. Specimen and sequencing details are provided in Table 1.

**Table 1.** Specimen and sequencing data for *Leucania comma*.

Project information			
**Study title**	Leucania comma		
**Umbrella BioProject**	PRJEB62567		
**Species**	*Leucania comma*		
**BioSample**	SAMEA112221976		
**NCBI taxonomy ID**	987968		
Specimen information			
**Technology**	**ToLID**	**BioSample ** **accession**	**Organism ** **part**
**PacBio long read sequencing**	ilLeuComm1	SAMEA112222383	head | thorax
**Hi-C sequencing**	ilLeuComm2	SAMEA112232909	head
**RNA sequencing**	ilLeuComm2	SAMEA112232911	abdomen
Sequencing information			
**Platform**	**Run ** **accession**	**Read count**	**Base count ** **(Gb)**
**Hi-C Illumina NovaSeq 6000**	ERR11496086	6.28e+08	94.86
**PacBio Sequel IIe**	ERR11483517	2.13e+06	25.9
**RNA Illumina NovaSeq X**	ERR12861035	6.52e+07	9.85

**Figure 2.**
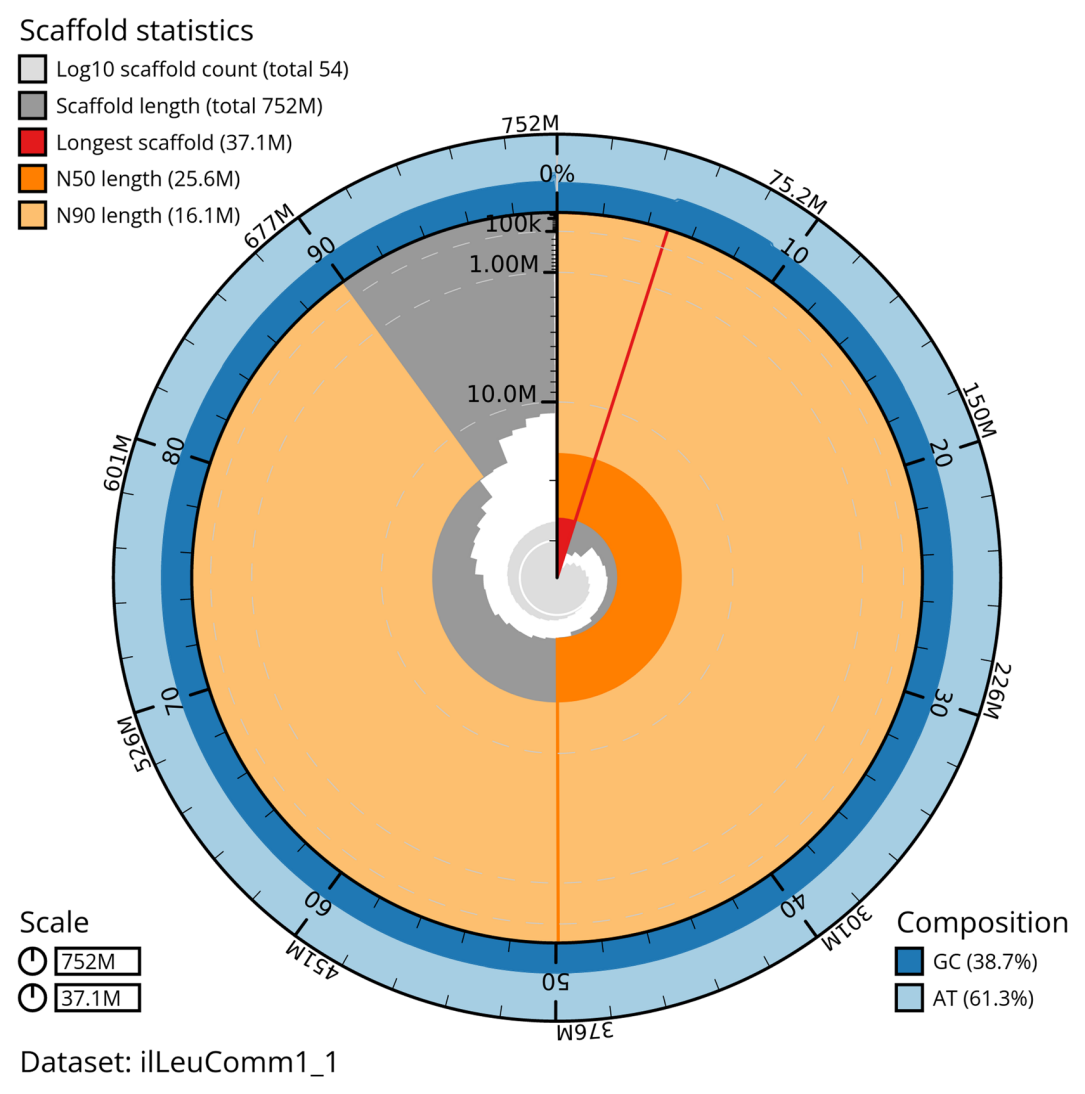
Genome assembly of *Leucania comma*, ilLeuComm1.1: metrics. The BlobToolKit snail plot shows N50 metrics and BUSCO gene completeness. The main plot is divided into 1,000 size-ordered bins around the circumference with each bin representing 0.1% of the 751,749,365 bp assembly. The distribution of scaffold lengths is shown in dark grey with the plot radius scaled to the longest scaffold present in the assembly (37,127,564 bp, shown in red). Orange and pale-orange arcs show the N50 and N90 scaffold lengths (25,646,328 and 16,099,744 bp), respectively. The pale grey spiral shows the cumulative scaffold count on a log scale with white scale lines showing successive orders of magnitude. The blue and pale-blue area around the outside of the plot shows the distribution of GC, AT and N percentages in the same bins as the inner plot. A summary of complete, fragmented, duplicated and missing BUSCO genes in the lepidoptera_odb10 set is shown in the top right. An interactive version of this figure is available at https://blobtoolkit.genomehubs.org/view/ilLeuComm1_1/dataset/ilLeuComm1_1/snail.

**Figure 3.**
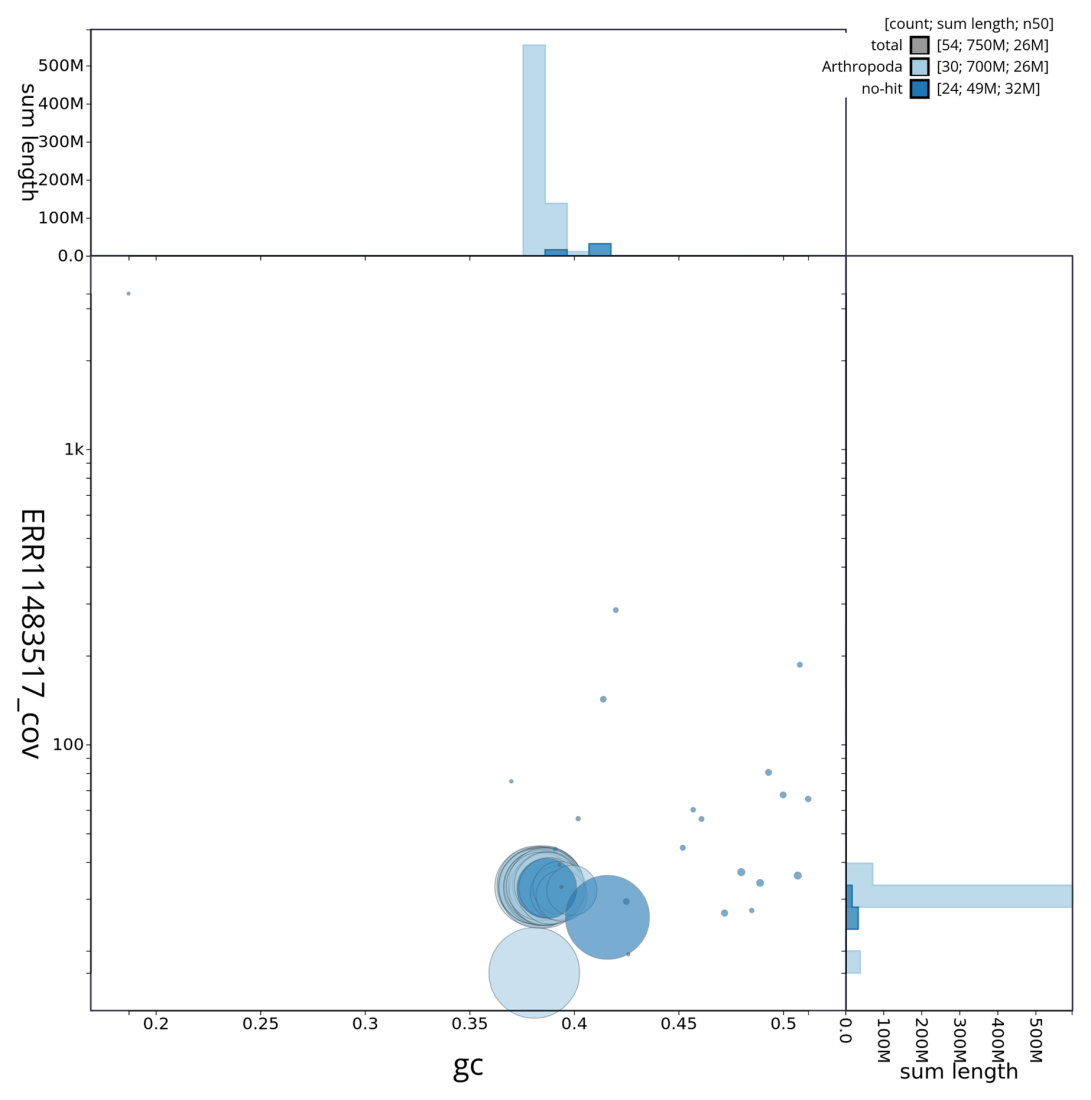
Genome assembly of *Leucania comma* ilLeuComm1.1: BlobToolKit blob plot. BlobToolKit GC-coverage plot showing sequence coverage (vertical axis) and GC content (horizontal axis). The circles represent scaffolds, with the size proportional to scaffold length and the colour representing phylum membership. The histograms along the axes display the total length of sequences distributed across different levels of coverage and GC content. An interactive version of this figure is available at https://blobtoolkit.genomehubs.org/view/ilLeuComm1_1/dataset/ilLeuComm1_1/blob.

**Figure 4.**
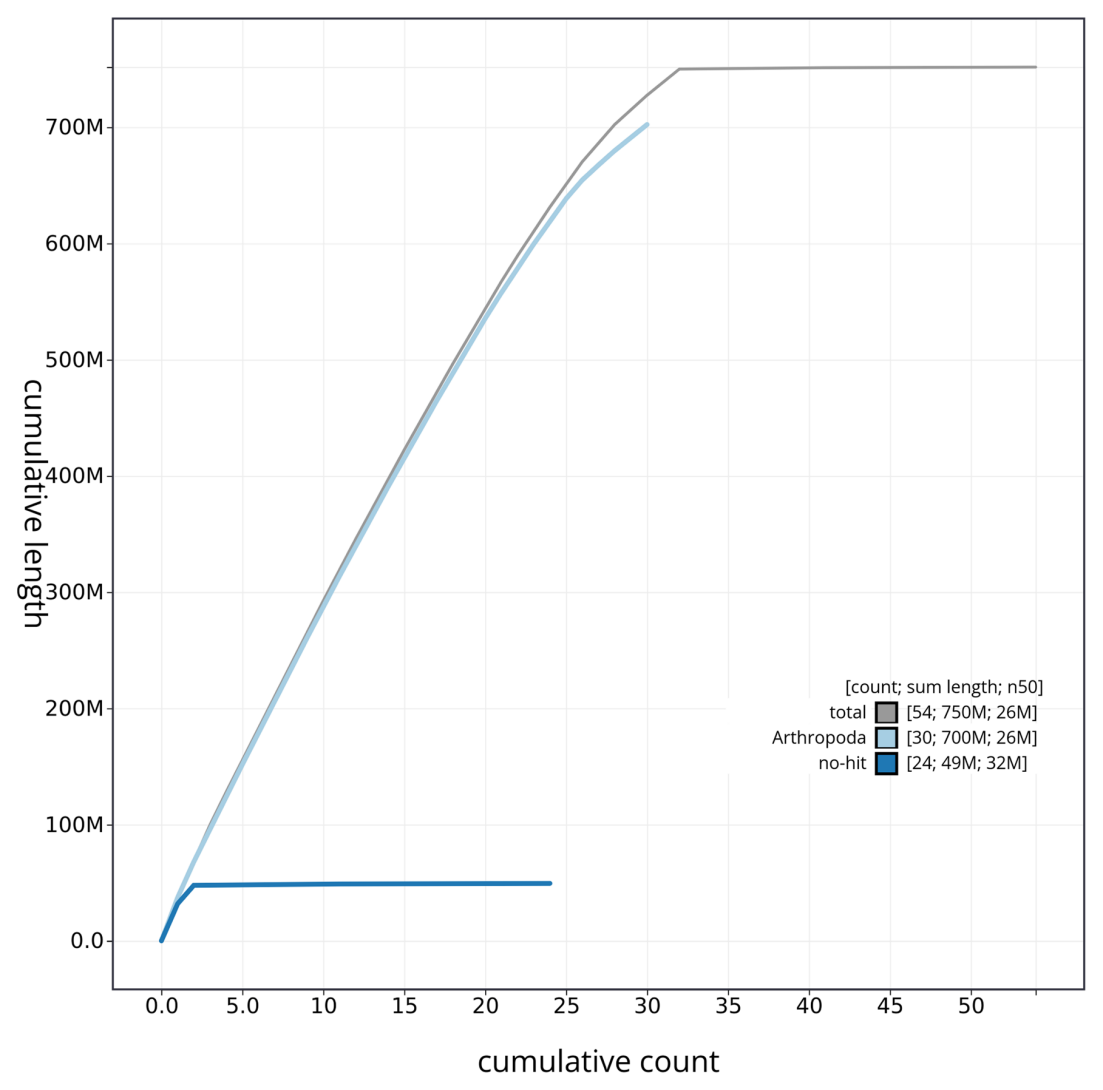
Genome assembly of *Leucania comma* ilLeuComm1.1: BlobToolKit cumulative sequence plot. The grey line shows cumulative length for all sequences. Coloured lines show cumulative lengths of sequences assigned to each phylum using the buscogenes taxrule. An interactive version of this figure is available at https://blobtoolkit.genomehubs.org/view/ilLeuComm1_1/dataset/ilLeuComm1_1/cumulative.

**Figure 5.**
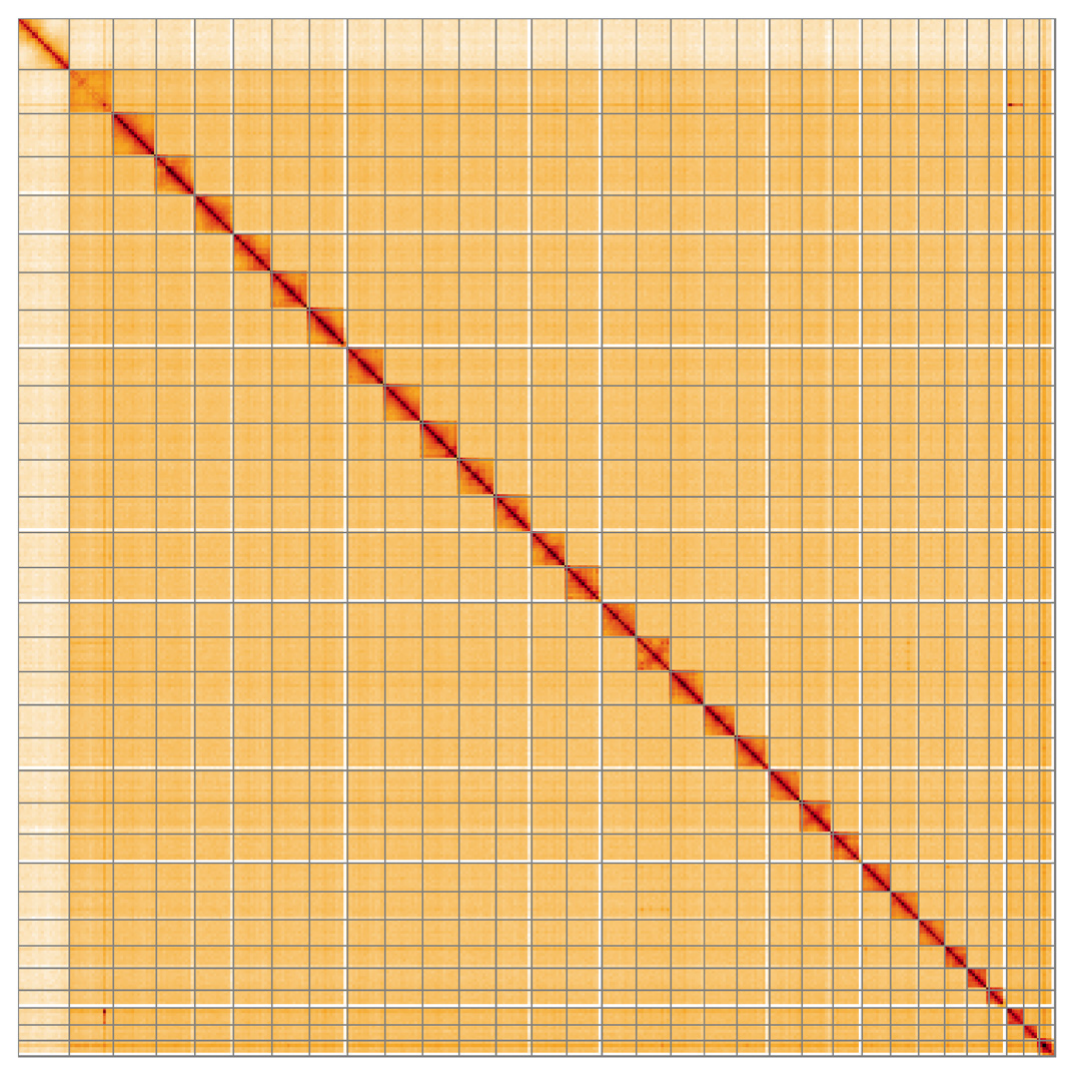
Genome assembly of *Leucania comma* ilLeuComm1.1: Hi-C contact map of the ilLeuComm1.1 assembly, visualised using HiGlass. Chromosomes are shown in order of size from left to right and top to bottom. An interactive version of this figure may be viewed at https://genome-note-higlass.tol.sanger.ac.uk/l/?d=T-rkejYsSKKTmIR4ebjEjw.

Manual assembly curation corrected 24 missing joins or mis-joins and five haplotypic duplications, reducing the scaffold number by 20.59%, and increasing the scaffold N50 by 1.08%. The final assembly has a total length of 751.70 Mb in 53 sequence scaffolds with a scaffold N50 of 25.6 Mb ( Table 2). The total count of gaps in the scaffolds is 102. The snail plot in Figure 2 provides a summary of the assembly statistics, while the distribution of assembly scaffolds on GC proportion and coverage is shown in Figure 3. The cumulative assembly plot in Figure 4 shows curves for subsets of scaffolds assigned to different phyla. Most (99.77%) of the assembly sequence was assigned to 32 chromosomal-level scaffolds, representing 30 autosomes and the Z and W sex chromosomes. Chromosome-scale scaffolds confirmed by the Hi-C data are named in order of size ( Figure 5; Table 3). Chromosomes Z and W were assigned based on read coverage statistics and synteny to *Lacanobia oleracea (*GCA_950371165.1) ( Davis *et al.*, 2024). While not fully phased, the assembly deposited is of one haplotype. Contigs corresponding to the second haplotype have also been deposited. The mitochondrial genome was also assembled and can be found as a contig within the multifasta file of the genome submission.

**Table 2.** Genome assembly data for *Leucania comma*, ilLeuComm1.1.

Genome assembly		
Assembly name	ilLeuComm1.1	
Assembly accession	GCA_958295575.1	
*Accession of alternate * *haplotype*	*GCA_958294965.1*	
Span (Mb)	751.70	
Number of contigs	156	
Number of scaffolds	53	
Longest scaffold (Mb)	37.13	
Assembly metrics *		*Benchmark*
Contig N50 length (Mb)	11.5	*≥ 1 Mb*
Scaffold N50 length (Mb)	25.6	*= chromosome N50*
Consensus quality (QV)	69.0	*≥ 40*
*k*-mer completeness	100.0%	*≥ 95%*
BUSCO **	C:99.0%[S:98.4%,D:0.6%], F:0.2%,M:0.7%,n:5,286	*S > 90%* *D < 5%*
Percentage of assembly mapped to chromosomes	99.77%	*≥ 90%*
Sex chromosomes	ZW	*localised homologous * *pairs*
Organelles	Mitochondrial genome: 15.37 kb	*complete single alleles*
Genome annotation of assembly GCA_958295575.1 at Ensembl		
Number of protein-coding genes	12,477	
Number of non-coding genes	1,858	
Number of gene transcripts	22,751	

* Assembly metric benchmarks are adapted from column VGP-2020 of “Table 1: Proposed standards and metrics for defining genome assembly quality” from Rhie *et al.* (2021). ** BUSCO scores based on the lepidoptera_odb10 BUSCO set using version 5.3.2. C = complete [S = single copy, D = duplicated], F = fragmented, M = missing, n = number of orthologues in comparison. A full set of BUSCO scores is available at https://blobtoolkit.genomehubs.org/view/ilLeuComm1_1/dataset/ilLeuComm1_1/busco.

**Table 3.** Chromosomal pseudomolecules in the genome assembly of *Leucania comma*, ilLeuComm1.

INSDC accession	Name	Length (Mb)	GC%
OY282463.1	1	30.82	38.5
OY282464.1	2	28.17	38.5
OY282465.1	3	27.98	38.0
OY282466.1	4	27.62	38.0
OY282467.1	5	27.39	38.5
OY282468.1	6	27.32	38.5
OY282469.1	7	27.26	38.5
OY282470.1	8	27.14	38.5
OY282471.1	9	26.53	38.5
OY282472.1	10	26.45	38.5
OY282473.1	11	25.81	38.0
OY282474.1	12	25.65	38.5
OY282475.1	13	25.37	38.0
OY282476.1	14	24.88	38.5
OY282477.1	15	24.8	38.5
OY282478.1	16	24.3	38.5
OY282479.1	17	23.67	38.5
OY282480.1	18	23.67	38.5
OY282481.1	19	23.49	38.5
OY282482.1	20	22.17	38.5
OY282483.1	21	21.09	38.5
OY282484.1	22	20.8	38.5
OY282485.1	23	20.09	39.0
OY282486.1	24	18.95	38.5
OY282487.1	25	16.1	39.0
OY282488.1	26	15.92	38.5
OY282489.1	27	12.87	39.0
OY282490.1	28	12.33	39.5
OY282491.1	29	11.4	39.5
OY282492.1	30	11.1	40.0
OY282462.1	W	31.81	41.5
OY282461.1	Z	37.13	38.0
OY282493.1	MT	0.02	19.0

The estimated Quality Value (QV) of the final assembly is 69.0 with *k*-mer completeness of 100.0%, and the assembly has a BUSCO v5.3.2 completeness of 99.0% (single = 98.4%, duplicated = 0.6%), using the lepidoptera_odb10 reference set ( *n* = 5,286).

Metadata for specimens, BOLD barcode results, spectra estimates, sequencing runs, contaminants and pre-curation assembly statistics are given at https://links.tol.sanger.ac.uk/species/987968.

## Genome annotation report

The *Leucania comma* genome assembly (GCA_958295575.1) was annotated at the European Bioinformatics Institute (EBI) on Ensembl Rapid Release. The resulting annotation includes 22,751 transcribed mRNAs from 12,477 protein-coding and 1,858 non-coding genes ( Table 2; https://rapid.ensembl.org/Leucania_comma_GCA_958295575.1/Info/Index). The average transcript length is 20,195.43. There are 1.59 coding transcripts per gene and 7.54 exons per transcript.

## Methods

### Sample acquisition and DNA barcoding

An adult female *Leucania comma* (specimen ID NHMUK014536952, ToLID ilLeuComm1) ( Figure 1) was collected from Gilbert White’s House, Selborne, England, UK (latitude 51.09, longitude –0.94) on 2021-06-10, using a light trap. The specimen was collected by a group from the Natural History Museum: Inez Januszczak, Gavin Broad, Laura Sivess, Stephanie Holt and Chris Fletcher. The specimen was formally identified by Stephanie Holt and then preserved by dry freezing at –80 °C.

The specimen used for Hi-C and RNA sequencing (specimen ID Ox002237, ToLID ilLeuComm2) was an adult specimen collected from Bratton, Somerset, UK (latitude 51.16, longitude –3.51) on 2022-06-20, using a light trap. The specimen was collected and identified by Denise Wawman (University of Oxford) and preserved on dry ice.

The initial species identification was verified by an additional DNA barcoding process according to the framework developed by Twyford *et al.* (2024). A small sample was dissected from the specimens and stored in ethanol, while the remaining parts of the specimen were shipped on dry ice to the Wellcome Sanger Institute (WSI). The tissue was lysed, the COI marker region was amplified by PCR, and amplicons were sequenced and compared to the BOLD database, confirming the species identification ( Crowley *et al.*, 2023). Following whole genome sequence generation, the relevant DNA barcode region is also used alongside the initial barcoding data for sample tracking at the WSI ( Twyford *et al.*, 2024). The standard operating procedures for Darwin Tree of Life barcoding have been deposited on protocols.io ( Beasley *et al.*, 2023).

### Nucleic acid extraction

The workflow for high molecular weight (HMW) DNA extraction at the WSI Tree of Life Core Laboratory includes a sequence of core procedures: sample preparation and homogenisation, DNA extraction, fragmentation and purification. Detailed protocols are available on protocols.io ( Denton *et al.*, 2023b). The ilLeuComm1 sample was weighed and dissected on dry ice ( Jay *et al.*, 2023) and tissue from the head and thorax was homogenised using a PowerMasher II tissue disruptor ( Denton *et al.*, 2023a).

HMW DNA was extracted in the WSI Scientific Operations core using the Automated MagAttract v2 protocol ( Oatley *et al.*, 2023). The DNA was sheared into an average fragment size of 12–20 kb in a Megaruptor 3 system ( Bates *et al.*, 2023). Sheared DNA was purified by solid-phase reversible immobilisation, using AMPure PB beads to eliminate shorter fragments and concentrate the DNA ( Strickland *et al.*, 2023). The concentration of the sheared and purified DNA was assessed using a Nanodrop spectrophotometer and Qubit Fluorometer using the Qubit dsDNA High Sensitivity Assay kit. Fragment size distribution was evaluated by running the sample on the FemtoPulse system.

RNA was extracted from abdomen tissue of ilLeuComm2 in the Tree of Life Laboratory at the WSI using the RNA Extraction: Automated MagMax™ *mir*Vana protocol ( do Amaral *et al.*, 2023). The RNA concentration was assessed using a Nanodrop spectrophotometer and a Qubit Fluorometer using the Qubit RNA Broad-Range Assay kit. Analysis of the integrity of the RNA was done using the Agilent RNA 6000 Pico Kit and Eukaryotic Total RNA assay.

### Hi-C preparation

Head tissue of the ilLeuComm2 sample was processed using the Arima-HiC v2 kit at the WSI Scientific Operations core. In brief, frozen tissue (stored at –80 °C) was fixed, and the DNA crosslinked using a TC buffer with 22% formaldehyde. After crosslinking, the tissue was homogenised using the Diagnocine Power Masher-II and BioMasher-II tubes and pestles. Following the kit manufacturer's instructions, crosslinked DNA was digested using a restriction enzyme master mix. The 5’-overhangs were then filled in and labelled with biotinylated nucleotides and proximally ligated. An overnight incubation was carried out for enzymes to digest remaining proteins and for crosslinks to reverse. A clean up was performed with SPRIselect beads prior to library preparation.

### Library preparation and sequencing

Library preparation and sequencing were performed at the WSI Scientific Operations core. Pacific Biosciences HiFi circular consensus DNA sequencing libraries were prepared using the PacBio Express Template Preparation Kit v2.0 (Pacific Biosciences, California, USA) as per the manufacturer’s instructions. The kit includes the reagents required for removal of single-strand overhangs, DNA damage repair, end repair/A-tailing, adapter ligation, and nuclease treatment. Library preparation also included a library purification step using 0.8X AMPure PB beads and a size selection step to remove templates < 3 kb using AMPure PB modified SPRI. Samples were sequenced using the Sequel IIe system (Pacific Biosciences, California, USA). The concentration of the library loaded onto the Sequel IIe was within the manufacturer's recommended loading concentration range of 40–100 pM. The SMRT link software, a PacBio web-based end-to-end workflow manager, was used to set-up and monitor the run, as well as perform primary and secondary analysis of the data upon completion.

For Hi-C library preparation, DNA was fragmented to a size of 400 to 600 bp using a Covaris E220 sonicator. The DNA was then enriched, barcoded, and amplified using the NEBNext Ultra II DNA Library Prep Kit following manufacturers’ instructions. The Hi-C sequencing was performed using paired-end sequencing with a read length of 150 bp on an Illumina NovaSeq 6000 instrument.

Poly(A) RNA-Seq libraries were constructed using the NEB Ultra II RNA Library Prep kit, following the manufacturer’s instructions. RNA sequencing was performed on the Illumina NovaSeq X instrument.

### Genome assembly, curation and evaluation

***Assembly***

The HiFi reads were first assembled using Hifiasm ( Cheng *et al.*, 2021) with the --primary option. Haplotypic duplications were identified and removed using purge_dups ( Guan *et al.*, 2020). The Hi-C reads were mapped to the primary contigs using bwa-mem2 ( Vasimuddin *et al.*, 2019). The contigs were further scaffolded using the provided Hi-C data ( Rao *et al.*, 2014) in YaHS ( Zhou *et al.*, 2023) using the --break option. The scaffolded assemblies were evaluated using Gfastats ( Formenti *et al.*, 2022), BUSCO ( Manni *et al.*, 2021) and MERQURY.FK ( Rhie *et al.*, 2020).

The mitochondrial genome was assembled using MitoHiFi ( Uliano-Silva *et al.*, 2023), which runs MitoFinder ( Allio *et al.*, 2020) and uses these annotations to select the final mitochondrial contig and to ensure the general quality of the sequence.

***Assembly curation***

The assembly was decontaminated using the Assembly Screen for Cobionts and Contaminants (ASCC) pipeline (article in preparation). Manual curation was primarily conducted using PretextView ( Harry, 2022), with additional insights provided by JBrowse2 ( Diesh *et al.*, 2023) and HiGlass ( Kerpedjiev *et al.*, 2018). Scaffolds were visually inspected and corrected as described by Howe *et al.* (2021). Any identified contamination, missed joins, and mis-joins were corrected, and duplicate sequences were tagged and removed. The sex chromosomes were assigned based on read coverage statistics and synteny analysis. The curation process is documented at https://gitlab.com/wtsi-grit/rapid-curation (article in preparation).

***Evaluation of the final assembly***

A Hi-C map for the final assembly was produced using bwa-mem2 ( Vasimuddin *et al.*, 2019) in the Cooler file format ( Abdennur & Mirny, 2020). To assess the assembly metrics, the *k*-mer completeness and QV consensus quality values were calculated in Merqury ( Rhie *et al.*, 2020). This work was done using the “sanger-tol/readmapping” ( Surana *et al.*, 2023a) and “sanger-tol/genomenote” ( Surana *et al.*, 2023b) pipelines. The genome assembly and evaluation pipelines were developed using nf-core tooling ( Ewels *et al.*, 2020) and MultiQC ( Ewels *et al.*, 2016), relying on the Conda package manager, the Bioconda initiative ( Grüning *et al.*, 2018), the Biocontainers infrastructure ( da Veiga Leprevost *et al.*, 2017), as well as the Docker ( Merkel, 2014) and Singularity ( Kurtzer *et al.*, 2017) containerisation solutions.

The genome was also analysed within the BlobToolKit environment ( Challis *et al.*, 2020) and BUSCO scores ( Manni *et al.*, 2021) were calculated.

Table 4 contains a list of relevant software tool versions and sources.

**Table 4.** Software tools: versions and sources.

Software tool	Version	Source
BlobToolKit	4.2.1	https://github.com/blobtoolkit/blobtoolkit
BUSCO	5.3.2	https://gitlab.com/ezlab/busco
bwa-mem2	2.2.1	https://github.com/bwa-mem2/bwa-mem2
Cooler	0.8.11	https://github.com/open2c/cooler
Gfastats	1.3.6	https://github.com/vgl-hub/gfastats
Hifiasm	0.16.1-r375	https://github.com/chhylp123/hifiasm
HiGlass	1.11.6	https://github.com/higlass/higlass
Merqury	MerquryFK	https://github.com/thegenemyers/MERQURY.FK
MitoHiFi	2	https://github.com/marcelauliano/MitoHiFi
PretextView	0.2	https://github.com/wtsi-hpag/PretextView
purge_dups	1.2.3	https://github.com/dfguan/purge_dups
sanger-tol/genomenote	v1.0	https://github.com/sanger-tol/genomenote
sanger-tol/readmapping	1.1.0	https://github.com/sanger-tol/readmapping/tree/1.1.0
Singularity	3.9.0	https://github.com/sylabs/singularity
YaHS	yahs-1.1.91eebc2	https://github.com/c-zhou/yahs

### Genome annotation

The Ensembl Genebuild annotation system ( Aken *et al.*, 2016) was used to generate annotation for the *Leucania comma* assembly (GCA_958295575.1) in Ensembl Rapid Release at the EBI. Annotation was created primarily through alignment of transcriptomic data to the genome, with gap filling via protein-to-genome alignments of a select set of proteins from UniProt ( UniProt Consortium, 2019).

### Wellcome Sanger Institute – Legal and Governance

The materials that have contributed to this genome note have been supplied by a Darwin Tree of Life Partner. The submission of materials by a Darwin Tree of Life Partner is subject to the **‘Darwin Tree of Life Project Sampling Code of Practice’**, which can be found in full on the Darwin Tree of Life website here. By agreeing with and signing up to the Sampling Code of Practice, the Darwin Tree of Life Partner agrees they will meet the legal and ethical requirements and standards set out within this document in respect of all samples acquired for, and supplied to, the Darwin Tree of Life Project.

Further, the Wellcome Sanger Institute employs a process whereby due diligence is carried out proportionate to the nature of the materials themselves, and the circumstances under which they have been/are to be collected and provided for use. The purpose of this is to address and mitigate any potential legal and/or ethical implications of receipt and use of the materials as part of the research project, and to ensure that in doing so we align with best practice wherever possible. The overarching areas of consideration are:

•   Ethical review of provenance and sourcing of the material

•   Legality of collection, transfer and use (national and international)

Each transfer of samples is further undertaken according to a Research Collaboration Agreement or Material Transfer Agreement entered into by the Darwin Tree of Life Partner, Genome Research Limited (operating as the Wellcome Sanger Institute), and in some circumstances other Darwin Tree of Life collaborators.

## Data Availability

European Nucleotide Archive: Leucania comma. Accession number PRJEB62567; https://identifiers.org/ena.embl/PRJEB62567. The genome sequence is released openly for reuse. The *Leucania comma* genome sequencing initiative is part of the Darwin Tree of Life (DToL) project. All raw sequence data and the assembly have been deposited in INSDC databases. The genome will be annotated using available RNA-Seq data and presented through the Ensembl pipeline at the European Bioinformatics Institute. Raw data and assembly accession identifiers are reported in Table 1 and Table 2.

## Author information

Members of the University of Oxford and Wytham Woods Genome Acquisition Lab are listed here: https://doi.org/10.5281/zenodo.12157525.

Members of the Natural History Museum Genome Acquisition Lab are listed here: https://doi.org/10.5281/zenodo.12159242.

Members of the Darwin Tree of Life Barcoding collective are listed here: https://doi.org/10.5281/zenodo.12158331.

Members of the Wellcome Sanger Institute Tree of Life Management, Samples and Laboratory team are listed here: https://doi.org/10.5281/zenodo.12162482.

Members of Wellcome Sanger Institute Scientific Operations: Sequencing Operations are listed here: https://doi.org/10.5281/zenodo.12165051.

Members of the Wellcome Sanger Institute Tree of Life Core Informatics team are listed here: https://doi.org/10.5281/zenodo.12160324.

Members of the Tree of Life Core Informatics collective are listed here: https://doi.org/10.5281/zenodo.12205391.

Members of the Darwin Tree of Life Consortium are listed here: https://doi.org/10.5281/zenodo.4783558.
