# Supplementary material for: The genome sequence of the Shoulder-striped Wainscot moth, Leucania comma Linnaeus, 1761
Source: Wellcome Open Res. 2024 Nov 5;9:652. [Version 1] doi: 10.12688/wellcomeopenres.23326.1 (PMC11632220; doi:10.12688/wellcomeopenres.23326.1)
Supplement: Peer review history

# Reviewer report: Reviewer response for version 1

**DOI:** 10.21956/wellcomeopenres.25709.r112650

**Published:** 2024-12-10

**Authors:** Fahad Alqahtani

**Affiliations:** 1 King Abdulaziz City for Science and Technology, Riyadh, Saudi Arabia

The authors of "The Genome Sequence of the Shoulder-striped Wainscot Moth, *Leucania comma* Linnaeus, 1761" have done a great job assembling a high-quality chromosome-level genome for an adult female *Leucania comma*. They used three sequencing technologies—PacBio long reads, Hi-C Illumina, and RNA sequencing—to build and annotate the assembly. The length of the final genome is 751.70 Mb and contains 32 chromosomes. In addition, they successfully constructed 15k bp mitogenome. The completeness of the assembly was assessed using BUSCO analysis, which showed that 99% of the genes from the lepidoptera_odb10 dataset are fully present.

 One suggestion for improving the paper would be to add a phylogenetic tree with related species. This would provide more context and make the study even more useful for comparison.

Are sufficient details of methods and materials provided to allow replication by others?

Yes

Is the rationale for creating the dataset(s) clearly described?

Yes

Are the datasets clearly presented in a useable and accessible format?

Yes

Are the protocols appropriate and is the work technically sound?

Yes

Reviewer Expertise:

Bioinformatics

I confirm that I have read this submission and believe that I have an appropriate level of expertise to confirm that it is of an acceptable scientific standard, however I have significant reservations, as outlined above.

# Reviewer report: Reviewer response for version 1

**DOI:** 10.21956/wellcomeopenres.25709.r112645

**Published:** 2024-12-09

**Authors:** Chris M Ward

**Affiliations:** 1 Australian Wine Research Institute, Urrbrae, South Australia, Australia

The article by Holt *et al *describes a chromosome level assembly of *Leucania comma*, the Shoulder-striped Wainscot moth. The genome adds to the growing number of genomes available for Lepidopterans and will be useful for future work on Lepidopteran pangenomics and *Leucania comma *conservation/population genomics.

 Although the assembly is a robust psuedo-haploid, a phase aware assembly method would improve the future applications of the genome in the pangenomics/gene hemizygosity space.

 Furthermore, the lack of RNAseq data derived from multiple life stages and/or tissue types may decrease the annotation and transcriptome completeness. 

 Other than these potential optimizations, the methods are sound and reproducible.

Are sufficient details of methods and materials provided to allow replication by others?

Yes

Is the rationale for creating the dataset(s) clearly described?

Yes

Are the datasets clearly presented in a useable and accessible format?

Yes

Are the protocols appropriate and is the work technically sound?

Yes

Reviewer Expertise:

Insect population genomics, Insect-Microbe interactions, Lepidopteran genome assembly and evolution, Plant pangenomics

I confirm that I have read this submission and believe that I have an appropriate level of expertise to confirm that it is of an acceptable scientific standard.

# Reviewer report: Reviewer response for version 1

**DOI:** 10.21956/wellcomeopenres.25709.r110231

**Published:** 2024-11-08

**Authors:** Christophe Klopp

**Affiliations:** 1 INRAE, Castanet-Tolosan, France

The authors present the first genome assembly of the Shoulder-striped Wainscot moth, Leucania comma *.*

 Having Hi-C and Hifi, the authors should have used the Hi-C reads during the hifiasm assembly in order to improve haplotype separation. 

 Publishing hap1 and hap2 rather than principal and alternative eases the downstream analysis such as pangenomics. This would have been a better option for hifiasm.

Are sufficient details of methods and materials provided to allow replication by others?

Yes

Is the rationale for creating the dataset(s) clearly described?

Yes

Are the datasets clearly presented in a useable and accessible format?

Yes

Are the protocols appropriate and is the work technically sound?

Partly

Reviewer Expertise:

Bioinformatics, genome assembly and annotation.

I confirm that I have read this submission and believe that I have an appropriate level of expertise to confirm that it is of an acceptable scientific standard, however I have significant reservations, as outlined above.

# Response

**Published:** 2024-11-08

**Authors:** Tree of Life Team Sanger

**Affiliations:** Wellcome Trust Sanger Institute, UK

Thank you for your detailed review and for highlighting the potential benefits of using Hi-C reads during the Hifiasm assembly process. We appreciate the value of incorporating Hi-C data for haplotype separation, and we have begun implementing this approach in recent projects to distinguish hap1 and hap2, rather than relying on primary and alternate representations. However, this particular assembly was generated in early 2023, at a time when using Hi-C data with Hifiasm for phasing was not yet part of our standard workflow. Additionally, the current assembly showed excellent continuity and completeness, leading us to focus on refining and curating a high-quality principal haplotype rather than explicitly phasing both haplotypes. The current assembly has been manually curated and refined, and it provides a valuable genomic resource for *Leucania comma*.
